# Assessment of the efficacy and safety of Ribavirin in treatment of coronavirus-related pneumonia (SARS, MERS and COVID-19): A protocol for systematic review and meta-analysis

**DOI:** 10.1097/MD.0000000000022379

**Published:** 2020-09-18

**Authors:** Yinhua Wang, Wen Li, Zhiming Jiang, Xiuming Xi, Yibing Zhu

**Affiliations:** a Department of Critical Care Medicine, Fuxing Hospital, Capital Medical University, Beijing; b Department of Critical Care Medicine, North China University of Science and Technology Affiliated Hospital, Tangshan; c Department of Critical Care Medicine, Peking University Third Hospital, Beijing; d Department of Respiratory Medicine, Qilu Hospital of Shandong University; e School of Medicine, Shandong University; f Department of Pulmonary and Critical Care Medicine, The First Affiliated Hospital of Shandong First Medical University, Jinan; g Medical Research and Biometrics Center, Fuwai Hospital, National Center for Cardiovascular Diseases, Chinese Academy of Medical Sciences and Peking Union Medical College, Beijing China.

**Keywords:** 2019 novel coronavirus disease, ribavirin, severe acute respiratory syndrome, Middle East respiratory syndrome, systematic review

## Abstract

**Background:**

The new coronavirus-related pneumonia is causing a global pandemic without specific antiviral drug. Ribavirin has activity against extensive RNA and DNA viruses. We plan to systematically review the use of ribavirin in patients with coronavirus-related pneumonia and meta-analyze the data with updated studies.

**Methods:**

EMBASE, PubMed, Cochrane Library, and China National Knowledge Infrastructure will be searched from 2002 to June 2021 without language restriction to identify randomized controlled trials. Subjects consist of patients with coronavirus-related pneumonia. Ribavirin of any dose or route will be compared with the control group of other medication, placebo, or no medication. The primary outcome is the hospital mortality. The secondary outcome includes the hospital length of stay, ventilator-free days in 28 days, median time from start of study treatment to negative nasopharyngeal swab, and adverse events. The Mantel-Haenszel method will be used for analysis of dichotomous data and the risk ratios will be reported with 95% confidence interval; the inverse-variance method will be used for continuous data and the mean differences will be reported. Sensitivity and subgroup analyses will be further performed. The funnel plots or Egger test will be used for detection of publication bias. The GRADE methodology will be used for summarizing the quality of evidence. The trial sequential analysis will be conducted to test whether the current meta-analysis is conclusive.

**Results:**

The efficacy and safety of ribavirin for treatment of coronavirus-related pneumonia will be systematically reviewed and summarized. The forthcoming results of the ongoing studies focusing on ribavirin in patients with the 2019 noel coronavirus disease will also be included.

**Conclusion:**

The relevant studies will be summarized and advanced evidence will be provided.

**PROSPERO registration number:**

CRD42020178900

## 1 Introduction

The new coronavirus-related pneumonia, COVID-19, is causing a severe public health emergency worldwide.^[1]^ However, no proved vaccines or antivirus treatment has been available so far.^[2,3]^ Ribavirin is a non–interferon-inducing antiviral agent, having activity against extensive RNA and DNA viruses.^[4]^ During the outbreak of severe acute respiratory syndrome (SARS) in 2003 and Middle East respiratory syndrome (MERS) in 2012, ribavirin was used for antivirus treatment in consideration of its broad-spectrum antiviral activity.^[5–7]^ The studies on ribavirin during SARS and MERS suggested a potential efficacy of using ribavirin; however, the quality of evidence remains low.^[7–11]^ With the previous experience and rationales, ribavirin has also been put into clinical practice in COVID-19. The National Health Commission of China recommended ribavirin intravenous infusion (500 mg per time, 2–3 times per day for <10 days, in combination with interferon or lopinavir/ritonavir) in the latest COVID-19 diagnosis and treatment plan (trial version 7).^[12]^ Through a search on the trail registration website of clinivaltrials.gov and Chinese Clinical Trail Registry (ChiCTR), we found several ongoing randomized controlled trials (RCTs). Therefore, we aim to systematically review the use of ribavirin on coronavirus-related pneumonia (SARS, MERSS, and COVID-19) and meta-analyze the data with the results of the updated RCTs to provide advanced evidence.

## 2 Review question

We aim to assess the safety and efficacy of Ribavirin in treatment of coronavirus-related pneumonia (SARS, MERS and COVID-19).

## 3 Methods

### 3.1 Study registration

This meta-analysis has been registered on the PROSPERO (registration number: CRD42020178900) on the PRISMA-P guideline.^[13]^

### 3.2 Search methods

Four electronic databases (PubMed, Cochrane Library, EMBASE, and China National Knowledge Infrastructure) will be searched to identify RCTs published from 2002 to June 2021 without language restriction. The potentially relevant references will also be searched. A search strategy using a combination of “coronavirus OR corona virus OR coronavirus-related OR SARS OR severe acute respiratory syndrome OR SARS-CoV MERS OR middle east respiratory syndrome OR MERS-CoV OR 2019nCoV OR COVID-19 OR SARS-CoV-2 OR novel coronavirus OR NCP” and “ribavirin OR virazole” in all fields has been developed. We also plan to re-run the search before the final analysis.

### 3.3 Inclusion criteria

#### 3.3.1 Studies

Only RCTs will be included in this analysis.

#### 3.3.2 Participants

The subjects consist of patients diagnosed with SARS, MERS, or COVID-19 of any age.

#### 3.3.3 Interventions/comparators

Ribavirin for the treatment of SARS, MERS, or COVID-19 of any dose or route will be compared with the control group of other medication, placebo, or no medication.

#### 3.3.4 Outcomes

The primary outcome is the hospital mortality. The secondary outcome includes the hospital length of stay, ventilator-free days in 28 days, median time from start of study treatment to negative nasopharyngeal swab, and adverse events.

### 3.4 Exclusion criteria

The studies only available as the abstract form will be excluded.

### 3.5 Data collection and analysis

#### 3.5.1 Study screening

Two reviewers (WY and LW) will independently screen the title/abstract of the records after removal of duplicates. The full text will be obtained for further assessment. The potentially relevant studies in the reference lists will also be searched. The flow diagram of selection process will be summarized for report.

#### 3.5.2 Data extraction

Two reviewers (WY and LW) will independently extract the data of first author, year of publication, study design, demographics of subjects, interventions, and outcomes of included studies to fill in a predesigned form. Any discrepancies will be resolved by a consulting group (XX and ZY).

#### 3.5.3 Assessment of study quality

Two reviewers (WY and LW) will independently evaluate the quality of included studies using the Cochrane Collaboration's tool.^[14]^ The quality of evidence will be assessed using the GRADE methodology.^[15,16]^ Any discrepancies will be solved by XX and ZY.

#### 3.5.4 Statistical analyses and data synthesis

Review Manager 5.3 will be applied for data analyses. For dichotomous data, the Mantel-Haenszel method will be used for the synthesis of risk ratios. For continuous data, the inverse-variance method will be used for the synthesis of mean differences. A 2-sided *P* value of <.05 will be considered statistically significant.

#### 3.5.5 Assessment of heterogeneity

A *χ*^2^ test and the statistic *I*^2^ will be used for assessment of heterogeneities.^[17]^ The *I*^2^ <40% suggests insignificant heterogeneity: 30% to 60% medium; 50% to 90% substantial; 76% to 100% high.^[17]^ Clinical and methodological heterogeneities will be assessed by the 2 reviewers (WY and LW) and discussed with the consulting group (XX and ZY) when needed. If there is no significant statistical, methodological, or clinical heterogeneity, a fixed-effect model will be used. Otherwise, a random-effect model will be chosen.^[17]^

#### 3.5.6 Subgroup and sensitivity analyses

Subgroup analyses of pneumonia caused by different viruses, different control groups, and different dosage or route of administration will be performed. The sensitivity analysis will be conducted by excluding each single study to test the robustness of the results.

#### 3.5.7 Assessment of publication bias

Funnel plots will be used to assess publication bias when there are >10 studies included. Otherwise, the Egger test will be used.^[18,19]^

#### 3.5.8 Trial sequential analysis

The trial sequential analysis (TSA) methodology will be used to assess whether the meta-analysis has a sufficient sample size to draw the current conclusion.^[20]^ The Copenhagen TSA software will be used for analyses.

## 4 Discussion

The in vitro studies showed an antiviral effect on SARS coronavirus (SARS-CoV) and MERS coronavirus (MERS-CoV).^[5,21,22]^ The pooled clinical data on ribavirin for treatment of SARS or MERS suggested a numerical higher survival rate without statistical significance.^[8–10]^ For safety concerns, there are studies suggesting that ribavirin might be associated with increased incidence of anemia and liver dysfunction.^[11,23–26]^ However, the evidence on SARS and MERS remains mostly based on cohort or case–control studies.^[8–11,27]^ For COVID-19, only in vitro data on the activity of ribavirin on SARS-CoV-2 are available so far.^[28]^ Till now the possible benefit and/or harm of ribavirin for treatment of coronavirus-related pneumonia are still inconclusive. Recently, the epidemic of COVID-19 remains serious in various regions and many clinical trials are designed to provide further evidence. Some ongoing RCTs focusing on ribavirin for COVID-19 can be identified on the registration websites. The forthcoming results of these RCTs will bring us a more comprehensive understanding on ribavirin and its indications. Our systematic review and meta-analysis will include these updated results and re-assess the efficacy and safety of ribavirin in patients with coronavirus-related pneumonia.

### 4.1 Uncited reference

^[12]^.

## Author contributions

**Conceptualization:** Yibing Zhu, Zhiming Jiang

**Data curation:** Yinhua Wang, Wen Li

**Methodology:** Xiuming Xi, Yibing Zhu

**Project administration:** Yibing Zhu

**Software:** Wen Li

**Supervision:** Xiuming Xi

**Writing – original draft:** Yinhua Wang, Wen Li

**Writing – review & editing:** Yibing Zhu, Xiuming Xi, Zhiming Jiang

## Abbreviations

Abbreviations: COVID-19 = 2019 noel coronavirus disease, MERS = Middle East respiratory syndrome, MERS-CoV = Middle East respiratory syndrome coronavirus, RCT = randomized controlled trial, SARS = severe acute respiratory syndrome, SARS-CoV = severe acute respiratory syndrome coronavirus, TSA = trial sequential analysis.
